# Exciton Modulation in Perylene-Based Molecular Crystals Upon Formation of a Metal-Organic Interface From Many-Body Perturbation Theory

**DOI:** 10.3389/fchem.2021.743391

**Published:** 2021-09-20

**Authors:** Liran Shunak, Olugbenga Adeniran, Guy Voscoboynik, Zhen-Fei Liu, Sivan Refaely-Abramson

**Affiliations:** ^1^ Department of Molecular Chemistry and Materials Science, Weizmann Institute of Science, Rehovot, Israel; ^2^ Department of Chemistry, Wayne State University, Detroit, MI, United States

**Keywords:** GW-BSE, Bethe–Salpeter equation, many-body perturbation theory (MBPT), exciton properties, metal-organic interface, molecular crystals, perylene diimide (PDI)

## Abstract

Excited-state processes at organic-inorganic interfaces consisting of molecular crystals are essential in energy conversion applications. While advances in experimental methods allow direct observation and detection of exciton transfer across such junctions, a detailed understanding of the underlying excitonic properties due to crystal packing and interface structure is still largely lacking. In this work, we use many-body perturbation theory to study structure-property relations of excitons in molecular crystals upon adsorption on a gold surface. We explore the case of the experimentally-studied octyl perylene diimide (C8-PDI) as a prototypical system, and use the GW and Bethe-Salpeter equation (BSE) approach to quantify the change in quasiparticle and exciton properties due to intermolecular and substrate screening. Our findings provide a close inspection of both local and environmental structural effects dominating the excitation energies and the exciton binding and nature, as well as their modulation upon the metal-organic interface composition.

Organic-inorganic interfaces play a key role in energy conversion and transfer processes (Wasielewski, 1992; Grätzel, 2001). Photoexcitations in the organic component typically generate bound electron and hole pairs, i.e., excitons, which serve as the main energy carriers and can effectively transfer energy across the interface (Ginley and Cahen, 2011). In particular, organic molecular crystals, composed of aromatic organic molecules bound together by van der Waals interactions (Klauk, 2006; Kronik and Neaton, 2016), are widely studied due to their easily adjustable characteristics and tunable excitonic properties (Sato et al., 1981; Smith and Michl, 2013; Luo et al., 2020), with relatively long diffusion lengths (Wan et al., 2015; Penwell et al., 2017; Schnedermann et al., 2019; Delor et al., 2020) stemming from their crystal structure, for example through singlet fission processes and the formation of long-lived triplet states (Wilson et al., 2013; Rao and Friend, 2017; Gish et al., 2019). The coupling between excitonic properties in molecular crystals and their underlying crystal packing and symmetry offers desirable tunability of their exciton relaxation processes and can lead to extended energy-transfer efficiency through material and interface design (Refaely-Abramson et al., 2017; Troisi and Orlandi, 2005; Cocchi et al., 2018; Arias et al., 2016; Cudazzo et al., 2012; Rangel et al., 2016). Of particular interest are the family of perylene diimide (PDI) molecular crystals, composed of a perylene body and an imide group and assembled by *π*-*π* interaction (Würthner et al., 2016; Schierl et al., 2018; Krieg et al., 2019; Santosh et al., 2010). Crystal packing and symmetry in PDI crystals vary strongly depending on their residues, allowing structural control of the exciton nature and diffusion length (Würthner et al., 2016; Schierl et al., 2018; Eaton et al., 2013; Zhang et al., 2018; Piland and Bardeen, 2015; Hestand and Spano, 2018; Oleson et al., 2019; Carter and Grossman, 2020; Wei et al., 2020), for example via a change in the imide substitution (Le et al., 2018; Felter et al., 2019). A commonly studied PDI crystal in organic optoelectronics is octyl-PDI (C8-PDI) (Felter et al., 2019; O’Brien et al., 2011; Krauss et al., 2009), shown in Figure 1A. The intermolecular interaction nature in this crystal gives rise to strongly bound excitons on one hand, and significant exciton dispersion on the other, making it a natural candidate for efficient exciton transfer upon formation of an organic-inorganic interface (Le et al., 2018; Cotton et al., 2020). In particular, C8-PDI single crystals and monolayers serve as a gate dielectric interface in working metal-organic devices, and the subtle details of interface design and structural inhomogeneity on charge and energy transfer efficiency within such junctions have been widely explored (Youn et al., 2012; Liscio et al., 2013; Ciccullo et al., 2015).

**FIGURE 1:**
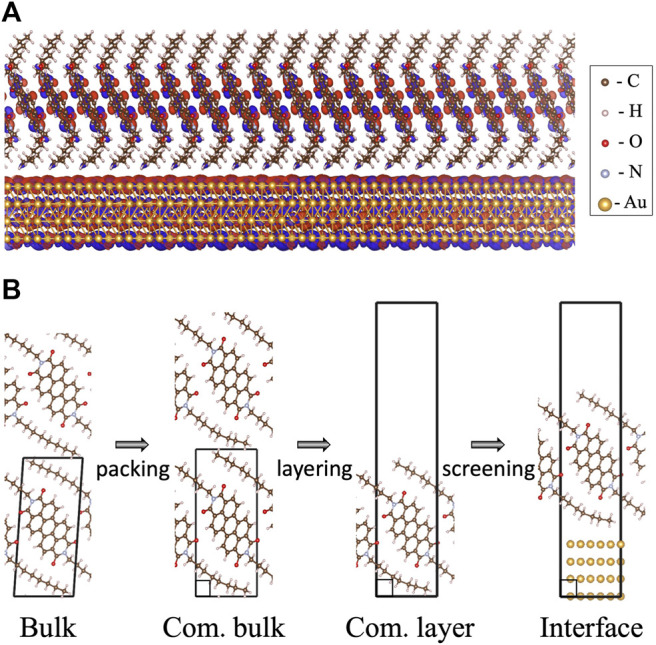
**(A)** The examined C8-PDI@Au metal-organic interface composed of an organic monolayer, commensurate with a four-layer Au (111) surface. The charge distribution computed for the lowest bound singlet exciton is also shown. **(B)** The different structural modifications of the interface compositions as examined in this work: C8-PDI intermolecular packing, layering, and adsorption on Au surface. The four main structures discussed in the text are termed as “bulk”, for the experimental triclinic crystal structure (Briseno et al., 2007); “com. bulk”, for an artificial construction of a commensurate phase; “com. layer”, for a monolayer structure of the commensurate phase; and “interface”, for an interface formed between the commensurate monolayer and four layers of Au (111).

These control capabilities at the atomistic level call for a computational examination of the change in excitonic properties stemming from the underlying structure, and in particular upon experimentally-accessible structural modifications. Such excitonic properties can be reliably computed using many-body perturbation theory (MBPT) within the GW and Bethe-Salpeter equation framework (GW-BSE) (Hybertsen and Louie, 1986; Rohlfing and Louie, 2000; Deslippe et al., 2012), a Green’s function based *ab initio* approach. GW-BSE computations have the predictive power required to relate the change in crystal packing to electronic and excitonic interaction nature. As such, this method has been applied in recent years to study quasiparticle and excitonic properties in bulk organic molecular crystals (Cudazzo et al., 2012; Sharifzadeh et al., 2012; Cudazzo et al., 2013; Sharifzadeh et al., 2013; Refaely-Abramson et al., 2015; Cudazzo et al., 2015; Kronik and Neaton, 2016; Rangel et al., 2016; Refaely-Abramson et al., 2017; Cocchi et al., 2018; Rangel et al., 2018). However, its explicit application on a metal-organic PDI interface is far from trivial, as it is highly computationally demanding. Nevertheless, such investigation can supply a comprehensive *ab initio* understanding of the relation between the structural changes at the various steps of interface construction - from the freestanding phase to an adsorbate on a metal surface - and the changes in quasiparticle and excitation properties dominating the energy transfer mechanisms.

In this study, we explore the change in excitonic properties in the C8-PDI organic molecular crystal upon structural modifications associated with metal-organic interface formation. For this, we investigate a series of systems, from the bulk molecular crystal with modified unit cells, through a monolayer structure, and finally an organic-inorganic interface formed by the adsorption of the C8-PDI monolayer on Au (111) surface. We use GW-BSE to study the quasiparticle and optical excitation energies in each system, and analyze the involved electron-hole binding as a function of intermolecular packing and layering. We demonstrate a close relationship between exciton localization and molecular arrangement in the crystal, manifesting the importance of intermolecular interactions beyond the Frenkel excitonic picture. We further investigate the effect of interlayer screening on the fundamental and optical gaps, and the resulting exciton binding energy. We show that while the quasiparticle energies are strongly affected by intermolecular and interlayer interactions, the optical excitation energies are far less sensitive to these structural modifications, demonstrating that the long-range nature of the interaction dominates the former and the short-range nature of the interaction dominates the latter. As a result, the computed exciton binding energy strongly depends on the crystal modifications. Our study presents an *ab initio* structure-sensitive understanding of excitonic properties in the C8-PDI@Au organic-inorganic interface, as a prototypical example which sheds light on the excited-state phenomena associated with the exciton transfer processes across such interfaces.

The paper is organized as follows: We first present the GW-BSE computational approaches used. Then we discuss the bulk C8-PDI quasiparticle and excitonic properties, emphasizing the exciton dispersion and the singlet and triplet state localization, and explore the effect of intermolecular packing upon unit cell modification to a commensurate structure. Finally, we examine variations in the excitonic picture in a freestanding layer, as opposed to the bulk structure, and upon adsorption on an Au substrate. The main steps in the structural modification explored are demonstrated in Figure 1B.

## 1 Computational Methods

We perform structural relaxation and compute initial electronic wavefunctions and energies, using density functional theory (DFT) (Kohn and Sham, 1965) and the Perdew-Burke-Ernzerhof (PBE) (Perdew et al., 1996) exchange-correlation functional, as implemented in the Quantum Espresso package (Giannozzi et al., 2017) (see full computational details in the SI). The DFT Kohn-Sham eigenvalues and eigenfunctions are then taken as the first guess for the MBPT calculations. We use the GW approximation to compute the quasiparticle energies and bandstructure (Hybertsen and Louie, 1986; Deslippe et al., 2012; Hedin, 1965), where we calculate self-energy corrections via Σ = *iGW*, for *G* the single-particle Green’s function and *W* the screened Coulomb interaction, $W_{GG^{\prime }}\left(q;0\right)=\varepsilon_{GG^{\prime }}^{-1}\left(q;0\right)v\left(q+G^{\prime }\right),$ where **G**, **G**′ are reciprocal lattice vectors, *v* is the bare Coulomb interaction, and *ɛ*_**GG**′_(**q**; 0) is the dielectric function of the system for interaction wavevector **q** and zero frequency, evaluated via (Hybertsen and Louie, 1986; Deslippe et al., 2012):

$$\varepsilon_{GG^{\prime }}\left(q;0\right)=\delta_{GG^{\prime }}-v\left(q+G\right)\chi_{GG^{\prime }}^{0}\left(q;0\right),$$ (1)

with $\chi_{GG^{\prime }}^{0}\left(q;0\right)$ the non-interacting electronic polarizability calculated using the random-phase approximation.

Optical excitations and excitonic properties are computed through the BSE formalism (Rohlfing and Louie, 1998; Rohlfing and Louie, 2000)

$$\begin{array}{c} \left(E_{ck+Q}-E_{vk}\right)A_{vckQ}^{S}+\underset{v^{\prime }c^{\prime }k^{\prime }}{\sum }\left\langle vk;ck+Q|K^{eh}|v^{\prime }k^{\prime };c^{\prime }k^{\prime }+Q\right\rangle A_{v^{\prime }c^{\prime }k^{\prime }Q}^{S}\\ =\Omega_{Q}^{S}A_{vckQ}^{S} \end{array}$$ (2)

for a hole state $\left|vk\rangle \right\rangle$ and an electron state $\left|ck+Q\rangle \right\rangle$, where **k** is the crystal momentum and **Q** is the exciton center-of-mass momentum. *S* indexes the exciton state at momentum **Q**. $A_{vckQ}^{S}$ is the amplitude of the free electron-hole pair. *E*_*c***k**+**Q**_ and *E*_*v***k**_ are the quasiparticle energies calculated within the GW approximation; $\Omega_{Q}^{S}$ is the excitation energy; and *K* ^*eh*^ is the electron-hole interaction kernel. Exciton dispersion is obtained by solving the BSE at different exciton momenta **Q** following the methodology developed in Refs. (Gatti and Sottile, 2013; Qiu et al., 2015). The GW-BSE exciton wavefunction can be represented as $\left|\Psi_{S,Q}\rangle \right\rangle =\sum_{vck}A_{vckQ}^{S}\left|ck+Q\rangle \right\rangle \left|vk\rangle \right\rangle$.

We compute the dielectric screening at the C8-PDI@Au interface using two approaches. The first is a direct calculation using G_0_W_0_ for the interface, which is highly computationally challenging and is made possible due to recent advances both in large-scale computing capabilities and associated code development (Del Ben et al., 2019). As a comparison, we also compute the dielectric function of the interface using a recently developed substrate screening GW approach (Liu et al., 2019). In this approach, the non-interacting polarizability of the interface is approximated by the summation of the separately calculated polarizabilies of the substrate (Au) and adsorbate (C8-PDI), i.e.,

$$\chi_{tot,GG^{\prime }}^{0}\left(q;0\right)\approx \chi_{mol,GG^{\prime }}^{0}\left(q;0\right)+\chi_{sub,GG^{\prime }}^{0}\left(q;0\right),$$ (3)

where $\chi_{\text{mol}}^{0}$ and $\chi_{\text{sub}}^{0}$ are the polarizabilities associated with the standalone molecular layer and metal substrates, respectively. This approximation holds for systems with weak hybridization between the adsorbate and the substrate (Liu et al., 2019; Xuan et al., 2019; Adeniran et al., 2020). The goal is to assess the validity of Eq. 3 against a direct GW calculation of the interface, to determine the nature of the surface effect on quasiparticle and exciton properties of the C8-PDI.

As shown in Figure 1B, we mainly study four different structures: (i) “bulk”, a bulk crystal in its experimentally resolved triclinic unit cell and relaxed atomic coordinates; (ii) “com. bulk”, a modified bulk structure in an orthorhombic lattice, so that it is commensurate with the lattice of the Au substrate; (iii) “com. layer”, a layered orthorhombic structure with large vacuum, taken as one molecular layer along the *c* axis from (ii); and (iv) “interface”, the C8-PDI monolayer from (iii) adsorbed on four layers of Au (111) surface, with each layer consisting of 2 × 3 Au atoms and an adsorption height of 3.18 Å. Such thickness of the simulated Au surface was previously found to sufficiently capture wavefunction hybridization and dielectric screening effects (Liu et al., 2017; Refaely-Abramson et al., 2019). We apply periodic boundary conditions to all structures, and fully relax the internal coordinates for each one of the structures. For (i) and (ii), the calculations were carried out using the converged parameters of a (8 × 4 × 4) k-point grid and 600 bands in the summation to compute the dielectric matrix, as well as a kinetic energy cutoff of 80 Ry and a dielectric cutoff of 10 Ry. The calculation of the absorption spectrum was carried out via an interpolation to a finer (12 × 6 × 6) k-point grid. For (iii) and (iv), the calculations used a k-point grid of (8 × 4 × 1) and 1,000 bands in the summation to compute the dielectric matrix, as well as the same cutoffs as (i) and (ii). (i)-(iii) used a semiconductor screening treatment of the **q** → 0 limit and (iv) used a metallic screening, as implemented in the BerkeleyGW package (Deslippe et al., 2012). Furthermore, (iii) and (iv) used a slab truncation for the Coulomb interaction.

## 2 Crystal Packing Effect on Exciton Nature

**FIGURE 2:**
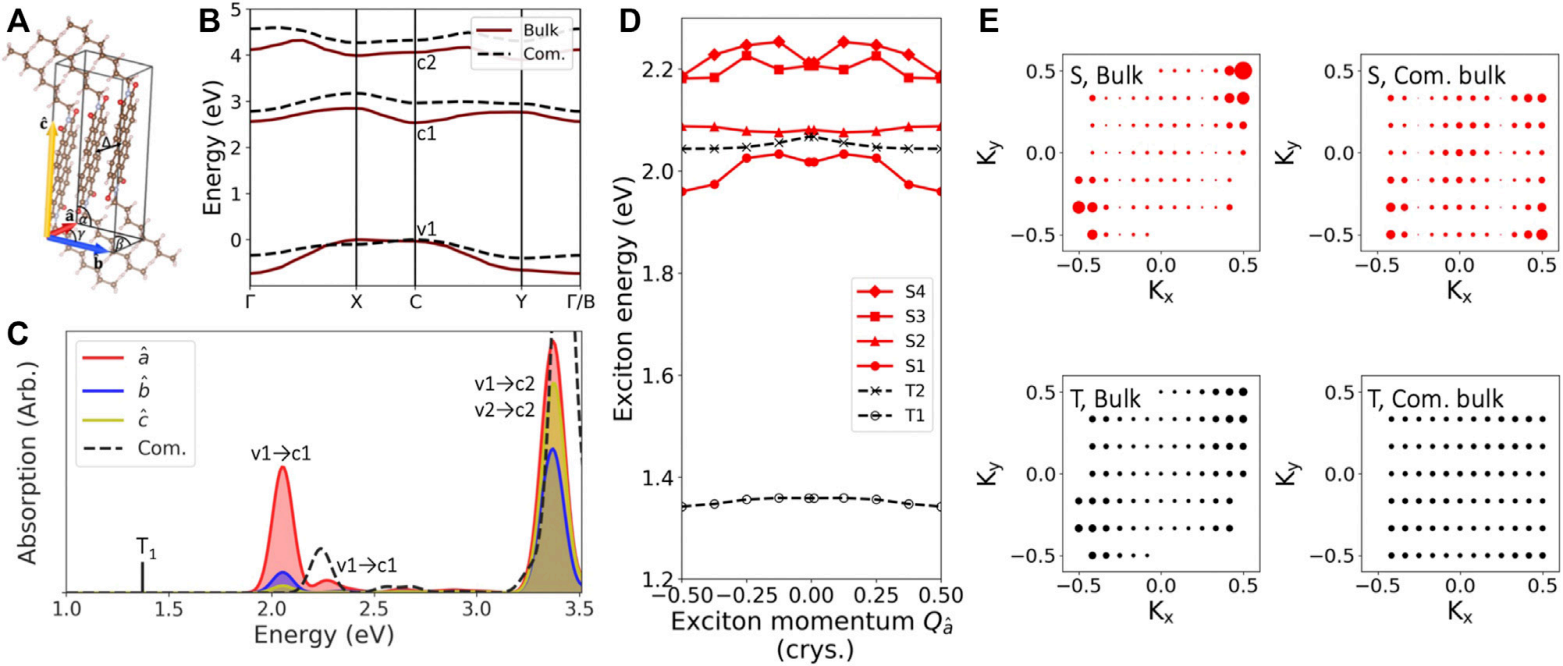
Structural and excitation properties of the bulk C8-PDI molecular crystal: **(A)** The unit cell showing the crystallographic parameters, which are modified among the various bulk structures examined. Δ denotes the intermolecular distance. **(B)** Computed GW quasiparticle bandstrcuture for the bulk (solid red) and the commensurate bulk (dashed black) structures. **(C)** Computed GW-BSE absorption spectra with the polarization of the light aligned along different cell directions. The low-lying triplet state is also shown (black line). Solid lines are for the bulk structure, and dashed black line is for the commensurate bulk structure. **(D)** Exciton bandstructure of the four lowest singlet states (solid red) and the two lowest triplet states (dashed black) of the bulk structure. **(E)** Exciton coefficients as a function of electron momentum in the (*K*_*x*_, *K*_*y*_) crystal plane for the lowest singlet and triplet states, of both the bulk and the commensurate bulk structures.

We begin with investigating the electronic and excitonic properties of the bulk C8-PDI system, using the experimentally-resolved crystal structure (Briseno et al., 2007), shown in Figure 2A (see full structural details in the SI). Figure 2B shows the computed GW quasiparticle bandstructure for this system (solid, red line). The three frontier electronic bands shown, valence (v_1_), conduction (c_1_), and second conduction (c_2_), dominate the low-lying excitonic spectra we discuss in this work. The computed GW quasiparticle gap is 2.6 eV, with an expected large self-energy correction on top of the DFT (PBE) gap of 1.1 eV. The quasiparticle bands are well isolated, as typical in molecular crystals due to the molecular-like nature of the material (Refaely-Abramson et al., 2013; Kronik and Neaton, 2016); still, we note a significant band dispersion of ∼0.7 eV for the valence band between k-points *X* and Γ and 0.3 eV for the conduction bands between *C* and *X*, reflecting non-negligible intermolecular electronic coupling.

To explore the effect of intermolecular orientation, we computed different bulk structures through variation of relative intermolecular distance and the crystallographic parameters of the unit cell (see SI for full details). Our motivation to vary these parameters is twofold: first, to explore the effect of molecular packing on the quasiparticles and excitons; second, to look into the effect of changes in unit cell vectors, which are needed to achieve proper commensurateness between the unit cell of the C8-PDI crystal and that of the Au surface. In order to maintain minimal strain within the Au surface, we only vary the PDI cell parameters. For the case of a single C8-PDI molecule per cell, this results in a modified cell (“com. bulk” in Figure 1B), in which the intermolecular distance grows compared to the original structure. Here we explore the limiting case of an orthorhombic molecular crystal unit cell, although a monoclinic cell leads to very similar results (see SI). By allowing more molecules per cell, commensurate layers can be achieved with smaller atomic modifications, however, performing GW-BSE calculations on these large supercells in an accurate manner is highly computationally challenging. The associated structures and the computed GW bandstructures for each of these cells in its bulk form are given in Supplementary Table S1; Supplementary Figure S1.

Since the electronic and excitonic states stem from the molecular building blocks, the effect of such unit-cell modification is not trivial and depends on the level of the electronic and excitonic wavefunction localization. Upon structural reorganization from the “bulk” structure to the “com. bulk” cell that is commensurate with the Au surface, the intermolecular distance increases from 3.4 Å to 3.6 Å, and the cell angle *γ* (Figure 2A) changes from 82^◦^ to 90^◦^. As a result, the computed GW quasiparticle gap increases by 0.4 eV and the band dispersion decreases by 0.35 eV, as shown in Figure 2B. Two other bulk structures with intermediate variations reveal a gradual modification between these values (see SI).

The strong structural dependence of the quasiparticle band dispersion reveals a somewhat surprisingly large effect of the molecular packing and orientation on the quasiparticle and exciton picture. It stems from intermolecular interactions and screening, which vary significantly with intermolecular distances and relative orientation. Figure 2C shows the computed BSE absorption spectra of the “bulk” structure, with a low-energy singlet peak at 2.05 eV, in good agreement with the experimental value of ∼2.2 eV (Le et al., 2018; Felter et al., 2019). The absorption spectra along the three main polarization directions are shown in different colors, with the main optically-active dipole transitions along the $\hat{a}$ axis, namely through the *π* − *π* stacking direction, and the least active direction along the *c* axis. The low-lying triplet exciton is found at 1.4 eV, shown with a black line. As a comparison, the absorption computed for the commensurate bulk structure is shown as well (dashed black line). As shown in Table 1, comparing the “com. bulk” structure with the “bulk” structure, the lowest singlet excitation energy increases by 0.19 eV; however, the lowest triplet excitation energy increases by only 0.06 eV.

**TABLE 1:** Quasiparticle and optical excitation energies from GW-BSE for the various structures examined. All energies are in eV.

System	Crystal	Com. bulk	Com. layer	Interface
Quasiparticle gap	2.57	2.97	3.40	2.94
Lowest singlet excitation	2.05	2.24	2.13	2.24
Lowest triplet excitation	1.37	1.43	1.33	1.52
Singlet binding energy	0.52	0.73	1.27	0.70

We further examine the effect of intermolecular coupling on the exciton nature by investigating the exciton dispersion, where the center-of-mass momentum **Q** represents the momentum difference between the hole and the excited electron, which is taken into account in the BSE, Eq. 2; (Qiu et al., 2015). Figure 2D shows the computed exciton bandstructure along the optically-active $\hat{a}$ direction (other crystal directions are shown in the SI). The exciton band shape varies along the reciprocal space due to indirect transitions occurring between the Γ and *X* points. At small exciton momentum **Q**, the lowest singlet exciton, *S*_1_, shows a parabolic behavior, with effective mass of 0.35*m*_*e*_ (for *m*_*e*_ the electron mass). Higher singlet excitons are also shown, revealing varying levels of localization. As expected, the low-lying triplet state has a larger effective mass of 2.7*m*_*e*_, supporting its higher degree of real-space localization, as typical in organic molecular crystals (Rangel et al., 2016; Refaely-Abramson et al., 2017).

Figure 2E shows the exciton coefficients ($A_{vck}^{S}$ in Eq. 2) for the **Q** = 0 transition as a function of quasiparticle momentum in the (K_*x*_ , K_*y*_) crystal plane. The lowest singlet state shows high localization in momentum space at the C point ([0.5,0.5,0] in reciprocal space), suggesting spatial delocalization. On the contrary, for the lowest triplet state, the exciton coefficients are nearly uniform within the Brillouin zone, pointing to spatial localization. In the commensurate bulk structure, the same singlet excitons are more spread in reciprocal space, reflecting increased real-space localization. The triplet state is localized in both structures and experiences a smaller change due to the structural modification, compared to the singlet state. The sizable differences between singlet and triplet localization is coupled to the short-range exchange in C8-PDI, dominating intermolecular electron-hole coupling (Rohlfing and Louie, 2000; Rangel et al., 2016). Upon changes in intermolecular packing, induced exciton localization leads to enhanced exchange interactions. Our results thus reveal strong state- and structure-dependence of the exciton nature in bulk C8-PDI, as an outcome of the intermolecular interactions dominating the electron-hole coupling.

## 3 Dimensionality and Interface Effects on Exciton Binding

Next, we study the effect of crystal layering and surface adsorption on the excitation energies. As shown in Table 1, for the commensurate structure, the quasiparticle gap of a freestanding C8-PDI layer increases by ∼0.4 eV, while both the singlet and triplet exciton energies decrease by ∼0.1 eV, compared to the commensurate bulk structure. This results in a significant increase of the exciton binding energy, an expected result due to the strong dimensionality effect on the dielectric screening, as we further elaborate in the discussion section below. From a computational point of view, constructing a monolayer is motivated by its subsequent adsorption on Au substrate within a computationally tractable periodic cell. In the following we directly compare the charged and neutral excitations of the freestanding layer with the adsorbed one, to gain direct insight into the Au screening effect on exciton binding.

To capture the dielectric screening at the C8-PDI@Au interface, we compare two approaches: an explicit GW calculation of the entire interface, with the simulation cell presented in Figure 1; and a substrate screening GW approach (Liu et al., 2019), in which the polarizabilities of the two parts of the interface are computed separately and then combined in the interface cell, as we discuss in the methods section. Our motivation is to verify the validity of the substrate screening approximation for the C8-PDI@Au interface. If this is true, we can conclude that the effect of the Au substrate merely provides a dielectric media that renormalizes the quasiparticle and excitation energies within the C8-PDI molecular layer, rather than altering the nature of the quasiparticle orbitals and excitons of the C8-PDI via orbital hybridization.

**FIGURE 3:**
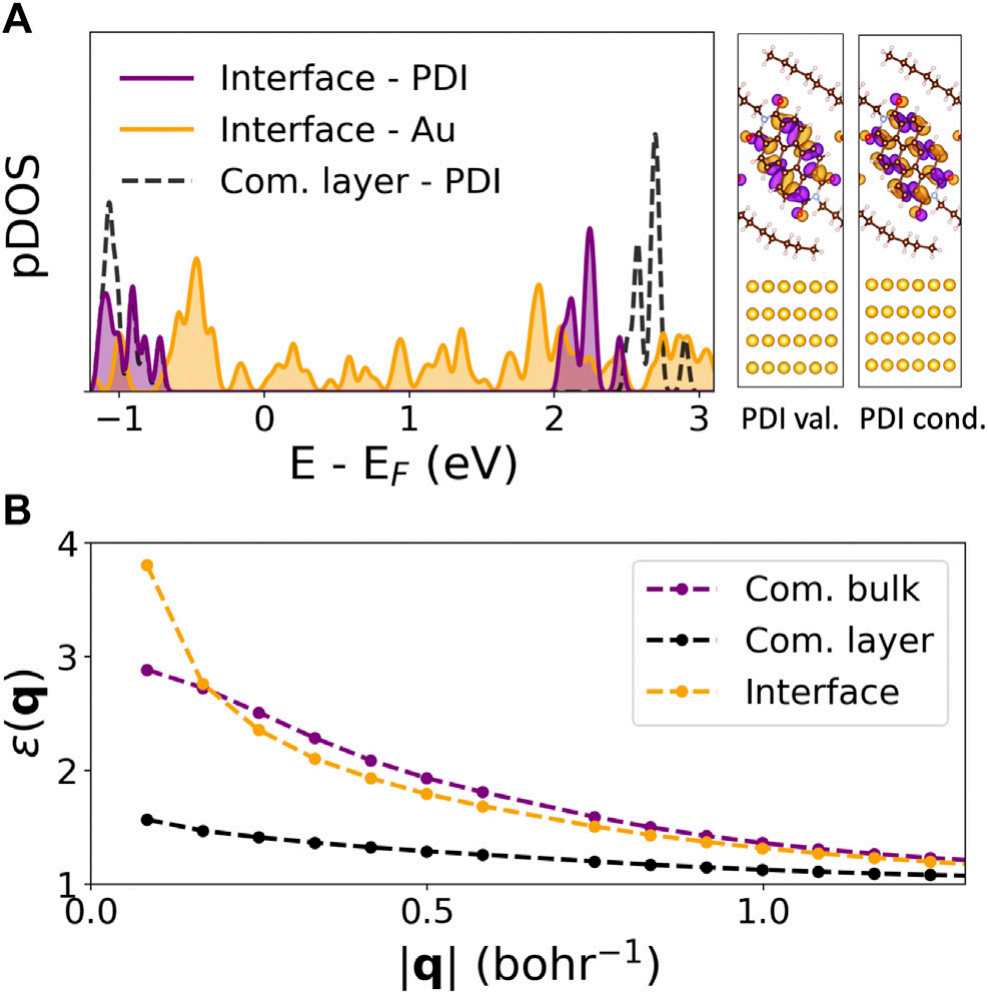
Quasiparticle and dielectric properties of the C8-PDI@Au interface: **(A)** GW pDOS on C8-PDI (purple) and Au (orange) atomic orbitals, compared to the freestanding molecular layer (dashed black). The right panel shows the electronic distributions of the PDI-localized valence and conduction states. **(B)** Computed GW dielectric function, *ɛ*(**q**), for the commensurate bulk (purple), commensurate layer (black), and the interface structure (orange).

Figure 3A shows the computed GW quasiparticle projected density of states (pDOS) of the full interface at high symmetry k-points onto C8-PDI (purple) and Au (orange) atomic orbitals. Dashed black lines represent the pDOS associated with the freestanding C8-PDI layer, which we align with the pDOS onto C8-PDI at the resonance corresponding to the highest occupied molecular orbital (at about −0.8 eV in Figure 3A). The quasiparticle gap associated with the molecular levels is reduced by 0.46 eV compared to the monolayer, due to the Au surface. The associated valence and conduction electronic charge distributions for the PDI-localized interface wavefunctions are well separated from the Au states, demonstrating the negligible interface hybridization in this system. The change in quasiparticle energies is a direct outcome of interface screening. Figure 3B compares the computed dielectric function *ɛ*_**00**_ (Eq. 1) of the commensurate bulk, layered, and interface systems for the head elements, namely **G** = **G**′ = 0. At large interaction distance **q**, corresponding to short-range interactions in real space, the dielectric function reaches the expected limit of *ɛ* = 1. At small **q**, however, the dielectric function increases significantly due to the Au screening, while remaining close to unity for the freestanding layer due to its reduced dimensionality.

The computed GW quasiparticle gap resulting from the substrate screening approach is 2.95 eV, which is in very good agreement with the direct calculation (2.94 eV). Additionally, in Supplementary Figure S5, we compare the diagonal elements of the non-interacting polarizability of the interface ($\chi_{\text{tot}}^{0}$) computed using the two approaches, and find that they agree very well. We can infer from these results that there is negligible orbital hybridization at the C8-PDI@Au interface, and that the Au substrate simply provides a dielectric environment that effectively screens the molecular crystal. We expect that the substrate screening approach, based on the additivity of the non-interacting polarizability of the interface (Liu et al., 2019; Xuan et al., 2019), is applicable to other systems without significant orbital hybridization or covalent bonding.

We believe this conclusion could help us understand the *physical* interface where a C8-PDI monolayer in its pristine bulk lattice (rather than the artificial commensurate lattice as we did in this work) is adsorbed on Au (111). From a computational perspective, such a physical interface is incommensurate and is prohibitively expensive to calculate. However, based on the conclusion achieved in this section, we can infer what would happen if we were modelling the incommensurate physical interface: all the properties associated to the exciton wavefunctions of the C8-PDI bulk crystal as reported in Figure 2 will be qualitatively unchanged in the interface, thanks to the negligible orbital hybridization. The exciton binding energies will be renormalized due to the dielectric screening of the Au substrate.

## 4 Discussion and Conclusion

**FIGURE 4:**
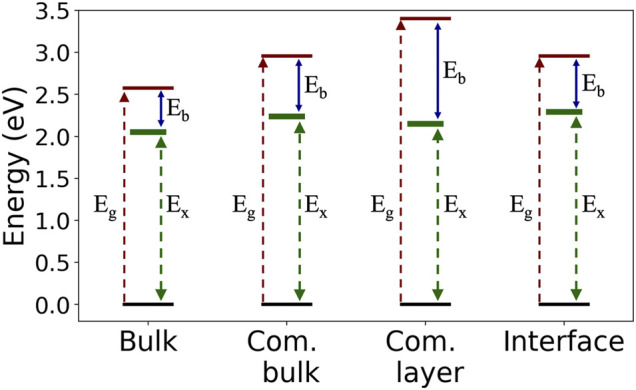
Summary of the computed GW quasiparticle gap, *E*_*g*_; optical gap, *E*_*x*_; and the exciton binding energy, *E*_*b*_, in the four examined structures.

Our results demonstrate the effect of intermolecular and surface screening on both quasiparticle and exciton properties via a step-by-step structural variation. Specifically, we look closely at the exciton nature as a function of molecular crystal packing and dielectric environment, as summarized in Figure 4. For the case of Frenkel-like molecular excitons, the exciton nature should stay roughly unchanged upon changes in crystal packing, as the environmental effect will be mainly manifested through an effective dielectric constant (Sharifzadeh et al., 2012; Cudazzo et al., 2013). Nonetheless, we find that the bulk C8-PDI excitons do have dispersion and are crystal-momentum dependent, reflecting non-negligible excitonic coupling beyond the Frenkel picture. The dispersion is more enhanced in singlet states compared to triplet states due to the exchange interaction, as observed before in related organic molecular crystals (Rangel et al., 2016; Refaely-Abramson et al., 2017; Rangel et al., 2018). On top of this effect, the dielectric screening induces further variations: upon crystal layering, the quasiparticle gap increases, and upon surface adsorption it renormalizes, while the exciton energies remain largely unchanged. This is a direct outcome of the non-local screening that dominates the quasiparticle energies, compared to local screening that dominates the electron-hole binding in the low-lying excitons. These dielectric effects are captured in the GW calculation through an explicit evaluation of the dielectric function (Hybertsen and Louie, 1986; Deslippe et al., 2012). As shown in Figure 3B, the dielectric functions of the various structures are similar at the short-range interaction regime and differ greatly at the long-range interaction regime.

Table 1 indicate the strong sensitivity of the charged quasiparticle excitation energies (as reflected in gaps) to the dielectric environment and the weak sensitivity of the neutral excitation energy (as reflected in optical transitions in the case of strong electron-hole coupling) to the environment (Kronik et al., 2012; Refaely-Abramson et al., 2015). The change in the optical gaps and the exciton binding energies between the C8-PDI monolayer and the C8-PDI@Au interface points to a significant decrease in the exciton binding energy upon formation of the interface with Au. This effect is expected, as the enhanced screening has a significant influence on the quasiparticle gap (Neaton et al., 2006; Egger et al., 2015; Liu et al., 2017). On the other hand, simple metal surfaces tend to have a much weaker influence on the optical excitations localized within the adsorbate (Spataru, 2013; Deilmann and Thygesen, 2019).

Importantly, the computational approach we employed here is not limited to cases of weak hybridization at the interface, and can be hence further used to investigate other types of PDI interfaces where the exciton separation mechanisms are expected to involve significant charge-transfer components. For example, triplet exciton transfer across C8-PDI and SiO_2_ interfaces was recently suggested to be strongly coupled to changes in intermolecular interactions due to surface hybridization (Cotton et al., 2020). In addition, few recent studies explored the role of surface passivation in modifying and controlling the efficiency of exciton transfer from acene molecular crystals adsorbed on silicon substrates (Einzinger et al., 2019; Daiber et al., 2020). The relation between interface bonding, charge and exciton localization far from and at the junction, and the resulting energy-transfer efficiency at PDI-based junctions is intriguing, and the results we present here for the case of weak metal-organic coupling can be thought of as a computational test case at the weakly interacting regime. Our results thus demonstrate that the GW-BSE approach offers a reliable tool to explore interface effects on excitonic properties at organic-inorganic interfaces with various levels of interface hybridization.

To conclude, we studied the effect of structural modifications on quasiparticle and exciton nature in the C8-PDI molecular crystal and its interface with Au. We explored the excitonic properties of the bulk system in detail, and investigated the effect of crystal packing and interface dielectric screening, building a structural modification route from the bulk structure to a heterogeneous interface. Our results demonstrate that while the quasiparticle band gap undergoes significant variations upon the structural modification and surface adsorption, the optical gap is much less affected by them, leading to strong structural sensitivity of the exciton binding energies. Our methods allow us to quantify this effect, and relate it to the specifics of local and non-local structural modifications.

## Data Availability

The datasets presented in this study can be found in online repositories. The names of the repository/repositories and accession number(s) can be found in the article/Supplementary Material.
