# Supplementary material for: Exciton Modulation in Perylene-Based Molecular Crystals Upon Formation of a Metal-Organic Interface From Many-Body Perturbation Theory
Source: Front Chem. 2021 Sep 20;9:743391. doi: 10.3389/fchem.2021.743391 (PMC8488370; doi:10.3389/fchem.2021.743391)
Supplement: Supplementary file 1 [file DataSheet1.PDF]

Supplemental Information: Exciton modulation in perylene-based molecular crystals upon formation of a metal-organic interface from many-body perturbation theory

Liran Shunak,[1] Olugbenga Adeniran,[2] Guy Voscoboynik,[1] Zhen-Fei Liu,[2] and Sivan Refaely-Abramson[1]

[1]*Department of Molecular Chemistry and Materials Science, Weizmann Institute of Science, Rehovot 7610001, Israel*

[2]*Department of Chemistry, Wayne State University, Detroit, Michigan 48202, United States*

1. Computational details

For the “bulk” C8-PDI structure, we use the experimentally reported crystallographic parameters [1] and relax the atomic positions using the Perdew-Burke-Ernzerhof (PBE) exchange-correlation functional [2] within density functional theory (DFT), as implemented in the Quantum Espresso package [3]. All the modified structures were relaxed in a similar manner, using fixed-cell atomic optimization. GW-BSE calculations were performed within the BerkeleyGW package [4]. For the bulk systems, GW calculations were carried out using a coarse k-point grid of $8\times4\times4$ and with 600 bands in the dielectric matrix, a kinetic energy cutoff of 80 Ry and a dielectric cutoff of 10 Ry. All parameters are converged within 0.1 eV in the quasiparticle energies. We construct the absorption spectrum by solving the Bethe-Salpeter equation (BSE), while interpolating the electron and hole states on a finer k-grid of $12\times6\times6$. For the layered structure of the orthorhombic system, a vacuum of 22.6 Å was added to the z direction of the cell, and the k-grid was reduced to $(8\times4\times1)$, with the absorption calculation interpolated on a fine grid of $(12\times6\times1)$. GW calculations of the C8-PDI@Au heterostructure were done on a coarse grid of $(8\times4\times1)$ with 1000 bands in the dielectric matrix and a metallic screening model as implemented in the BerkeleyGW package [4]. All GW calculations were performed using the generalized plasmon-pole approximation for the quasiparticle self-energy [5].

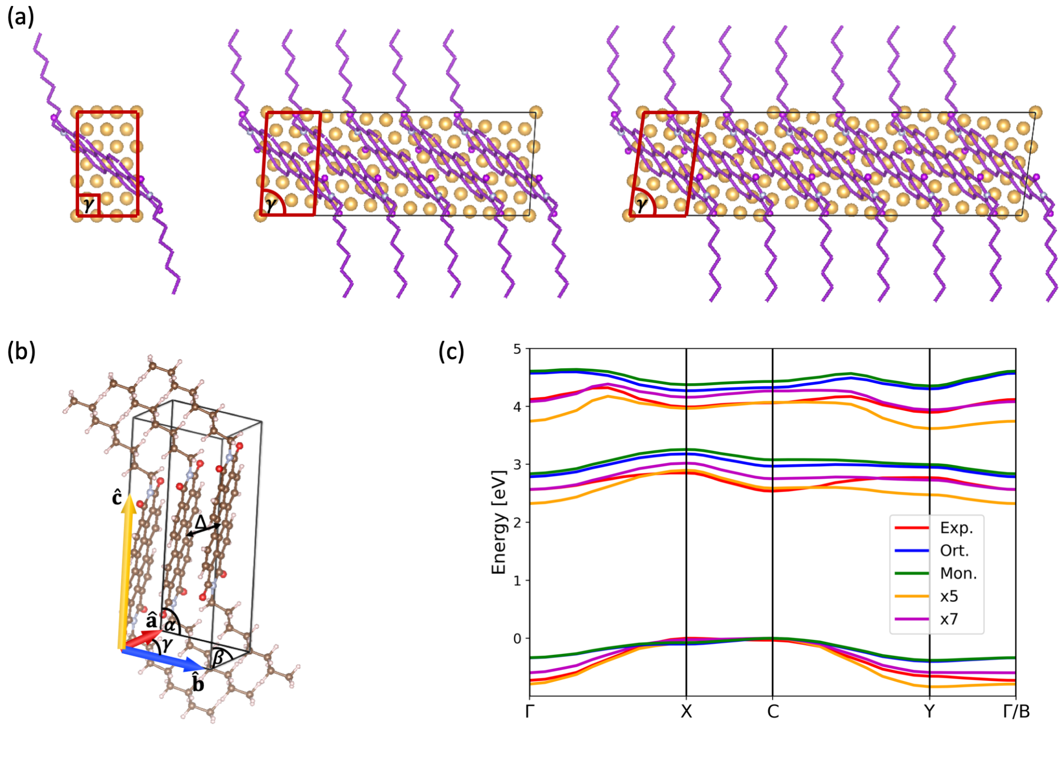

FIG. S1. (a) Top view of the various supercells with different number of C8-PDI unit cells (marked in red frame) on Au and the change in the angle γ, showing the ’Ort.’, ’$\times5$’, and ’$\times7$’ structures (from left to right). (b) Unit cell structural parameters of bulk C8-PDI, including the cell vectors and angles and the intermolecular distance Δ. (c) Computed GW bandstructures of the five different bulk systems: ’Exp.’, the experimental bulk structure; ’Ort.’ and ’Mon.’ for the modified orthorhombic and monoclinic cells, respectively; and ’$\times5$’, ’$\times7$’ for the extended super-cells showed in (a).

parameter	**Bulk**	**Ort.**	**Mon.**	**×5**	**×7**
a [Å]	4.67	4.99	4.99	4.50	4.68
b [Å]	8.50	8.65	8.65	8.65	8.65
c [Å]	19.72	19.42	19.72	19.72	19.72
α [°]	86	90	86	90	90
β [°]	88	90	88	90	90
γ [°]	83	90	90	86	82
Δ [Å]	3.39	3.45	3.44	3.37	3.58
Direct GW gap [eV]	2.57	3.07	3.08	2.58	2.75
Valence GW band dispersion [eV]	0.69	0.34	0.34	0.79	0.59

TABLE S1. Cell parameters for the original and modified unit cells, and GW results for the enregy band gap and the band dispersion.

2. Packing effect on GW bandstructure

We explored the effect of intermolecular orientation on the quasiparticle and excitonic properties within five different bulk structures, through variation of unit cell angles and consequently the relative intermolecular distance and alignment. The five structures contain (i) the experimental structure discussed above, which we mark as structure 'bulk'; (ii) an orthorhombic cell in which all angles are taken to be 90°, 'ort.'; (iii) a monoclinic cell, which conserves the angle between PDI layers but sets a 90° angle in the molecular plane, 'mon.'; in both (ii) and (iii), a single unit cell is commensurate with the Au surface. Two additional cells, '×5' and '×7', show the cases in which a super-cell of five and seven C8-PDI molecules, respectively, are used in order to achieve commensurateness with the Au layer as well. Fig. S1(a) presents a top view of these C8-PDI@Au super-cells, with the α and β angles kept at 90° and varying γ angle. We relaxed the atomic coordinates using DFT as described in the computational details above, while including the Au atoms in the system. All the cells parameters are presented in Table S1 and a scheme of the different parameters is given in Fig. S1(b).

3. Exciton dispersion and localization in bulk and layered C8-PDI

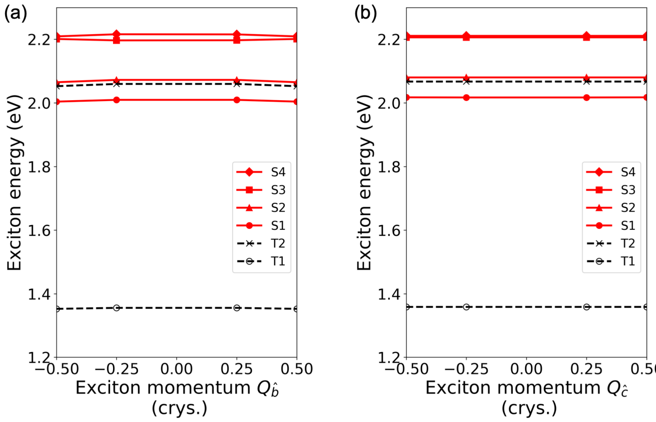

FIG. S2. Exciton dispersion in bulk C8-PDI (a) along $\hat{b}$ direction and (b) along $\hat{c}$ direction

GW quasiparticle bandstructures were computed for each system on the bulk C8-PDI unit cell, namely without including the Au layers and with a single molecule per repeating unit cell. The resulting GW bandstructures are given in Fig. S1(c), and the dispersion and quasiparticle gaps are listed in Table S1. Several immediate effects are noticeable: first, the valence band dispersion strongly depends on the intermolecular orientation; however, it only weakly depends on the interlayer orientation. This is expected, and results from local intermolecular interactions. The valence band dispersion of the ort. and mon. structures is smaller than the others, suggesting weaker interactions and induced localization in these structures; this is also supported by the increased quasiparticle gap. Both structures have almost identical bandstructure, manifesting the layer-like nature of the electronic (and excitonic) interactions in them. As the intermolecular orientation is retrieved through angle modification in the ×5 and ×7 structures, the computed dispersion increases and is similar to the original cell. Another important change is the conduction band minimum position, which alters from the C point to the Γ point upon orthorhombic cell construction. This is a

direct result of the change in wavefunction nature and localization. Overall, the examined cell modifications introduce significant changes in the intermolecular interactions, inducing increased wavefunction localization, and resulting with quasiparticle band dispersion and bandgap variations across the Brillouin Zone.

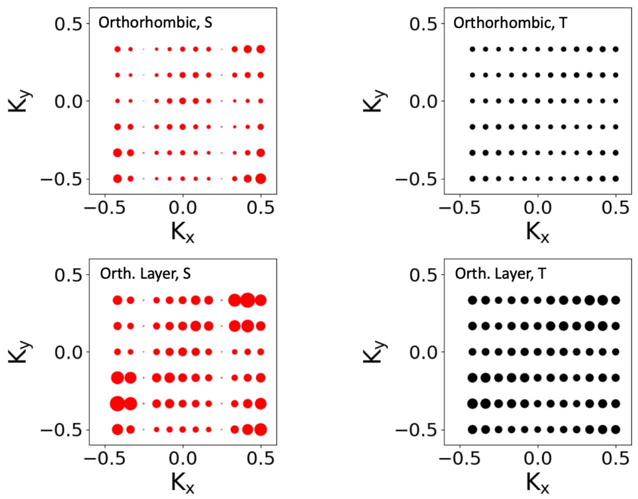

FIG. S3. Exciton coefficients as a function of electron momentum in the (K_x, K_y) crystal plane for the lowest singlet and triplet states, of orthorhombic bulk and layer structures.

Figure S2 shows the exciton energy as a function of momentum Q at the $\hat{b}$ and $\hat{c}$ lattice direction for the bulk C8-PDI system. The weak dispersion manifest these directions do not dominate the excitonic picture, and the excitons are roughly of molecular nature along them. Figure S3 shows the k-resolved exciton nature for the lowest singlet and triplet states of the orthorhombic bulk (top) and layered (bottom) structures. The two systems show similar exciton nature.

4. DFT interface bandstructure

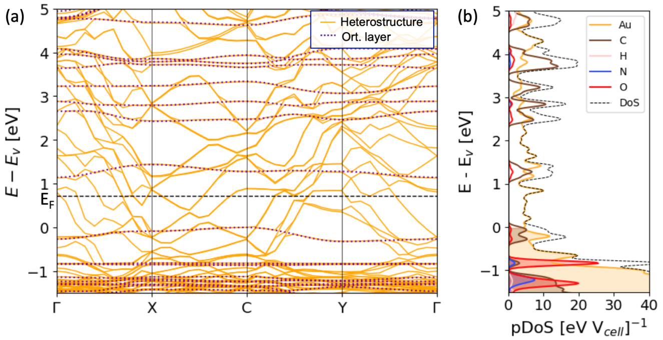

FIG. S4. DFT results for the interface system: (a) electronic bandstructure, (b) the projected density of state of different atoms around the Fermi energy from DFT.

Figure S4 shows the computed DFT bandstructure (a) and the associated projected density of states (pDOS) (b) for the C8-PDI@Au interface. The flat bands near the Fermi energy are identified as the PDI molecular-like bands, as manifested by the pDOS. The charge distribution of the C8-PDI localized states is reflected in flat bands, identical to those of the free-standing layer (dashed red lines in Fig. S4(a)).

5. Comparison of interface polarizabilities computed from direct GW and the substrate screening GW

Figure S5 shows the comparison between the polarizability matrices computed from direct GW and the substrate screening GW approach.

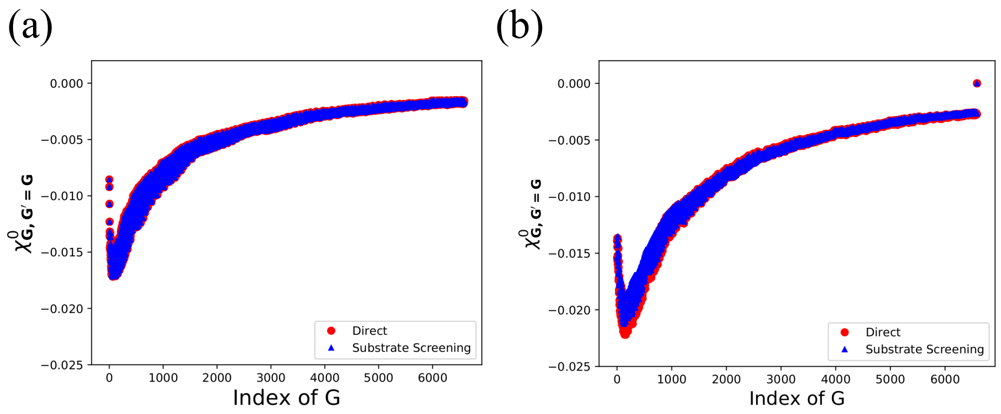

FIG. S5. Comparison of the diagonal elements of the non-interacting (Kohn-Sham) polarizability matrices computed from direct GW (red circle) and the substrate screening GW approach (blue triangle). This comparison is carried out at (a) the limit of $\mathbf{q} \rightarrow 0$; and (b) the high symmetry C-point in reciprocal space, with coordinate (0.5,0.5,0.0). In both panels, the horizontal axis is the row/column index of the χ^0 matrix.

[1] A. L. Briseno, S. C. Mannsfeld, C. Reese, J. M. Hancock, Y. Xiong, S. A. Jenekhe, Z. Bao, and Y. Xia, Nano Lett. **7**, 2847 (2007).
[2] J. P. Perdew, M. Ernzerhof, and K. Burke, J. Chem. Phys. **105**, 9982 (1996).
[3] P. Giannozzi, O. Andreussi, T. Brumme, O. Bunau, M. B. Nardelli, M. Calandra, R. Car, C. Cavazzoni, D. Ceresoli, M. Cococcioni, N. Colonna, I. Carnimeo, A. D. Corso, S. de Gironcoli, P. Delugas, R. A. D. Jr, A. Ferretti, A. Floris, G. Fratesi, G. Fugallo, R. Gebauer, U. Gerstmann, F. Giustino, T. Gorni, J. Jia, M. Kawamura, H.-Y. Ko, A. Kokalj, E. Küçükbenli, M. Lazzeri, M. Marsili, N. Marzari, F. Mauri, N. L. Nguyen, H.-V. Nguyen, A. O. de-la Roza, L. Paulatto, S. Poncé, D. Rocca, R. Sabatini, B. Santra, M. Schlipf, A. P. Seitsonen, A. Smogunov, I. Timrov, T. Thonhauser, P. Umari, N. Vast, X. Wu, and S. Baroni, J. Phys: Cond. Matt. **29**, 465901 (2017).
[4] J. Deslippe, G. Samsonidze, D. A. Strubbe, M. Jain, M. L. Cohen, and S. G. Louie, Comput. Phys. Commun **183**, 1269 (2012).
[5] M. S. Hybertsen and S. G. Louie, Phys. Chem. Chem. Phys **34**.
